# Transcription Profiles of Endothelial Cells in the Rat Ductus Arteriosus during a Perinatal Period

**DOI:** 10.1371/journal.pone.0073685

**Published:** 2013-09-27

**Authors:** Norika Mengchia Liu, Tomohiro Yokota, Shun Maekawa, Ping Lü, Inbun Tei, Hideki Taniguchi, Utako Yokoyama, Takashi Kato, Susumu Minamisawa

**Affiliations:** 1 Department of Life Science and Medical Bioscience, Waseda University, Tokyo, Japan; 2 Department of Biology School of Education, Waseda University, Tokyo, Japan; 3 Department of Cell Physiology, Jikei University, Tokyo Japan; 4 Department of Regenerative Medicine, Yokohama City University, Yokohama, Japan; 5 Cardiovascular Research Institute, Yokohama City University, Yokohama, Japan; UT-Southwestern Med Ctr, United States of America

## Abstract

Endothelial cells (ECs) lining the blood vessels serve a variety of functions and play a central role in the homeostasis of the circulatory system. Since the ductus arteriosus (DA) has different arterial characteristics from its connecting vessels, we hypothesized that ECs of the DA exhibited a unique gene profile involved in the regulation of DA-specific morphology and function. Using a fluorescence-activated cell sorter, we isolated ECs from pooled tissues from the DA or the descending aorta of Wistar rat fetuses at full-term of gestation (F group) or neonates 30 minutes after birth (N group). Using anti-CD31 and anti-CD45 antibodies as cell surface markers for ECs and hematopoietic derived cells, respectively, cDNAs from the CD31-positive and CD45-negative cells were hybridized to the Affymetrix GeneChip® Rat Gene 1.0 ST Array. Among 26,469 gene-level probe sets, 82 genes in the F group and 81 genes in the N group were expressed at higher levels in DA ECs than in aortic ECs (*p*<0.05, fold change>2.0). In addition to well-known endothelium-enriched genes such as Tgfb2 and Vegfa, novel DA endothelium-dominant genes including Slc38a1, Capn6, and Lrat were discovered. Enrichment analysis using GeneGo MetaCore software showed that DA endothelium-related biological processes were involved in morphogenesis and development. We identified many overlapping genes in each process including neural crest-related genes (Hoxa1, Hoxa4, and Hand2, etc) and the second heart field-related genes (Tbx1, Isl1, and Fgf10, etc). Moreover, we found that regulation of epithelial-to-mesenchymal transition, cell adhesion, and retinol metabolism are the active pathways involved in the network via potential interactions with many of the identified genes to form DA-specific endothelia. In conclusion, the present study uncovered several significant differences of the transcriptional profile between the DA and aortic ECs. Newly identified DA endothelium-dominant genes may play an important role in DA-specific functional and morphologic characteristics.

## Introduction

A wide variety of vascular biology studies in the past several decades have verified that the vascular endothelium plays a critical role in the homeostasis of the cardiovascular system. Endothelial cells (ECs) lining blood vessels form monolayers to cover the inner lumen of all types of the vessels in the normal state. The vascular endothelium regulates vasomotor tone by secretion of several vasoactive substances such as nitric oxide. It also mediates leukocyte trafficking and monocyte activation to control platelet adhesion and coagulation. In addition, it interacts with vascular smooth muscle to mediate proliferation and differentiation of smooth muscle cells (SMCs). ECs respond diversely to a variety of external or internal stimuli to alter membrane permeability, transcellular transport systems, membrane adhesive molecules, various growth factor secretions, and so on [1], [2]. These responses occur in order to fulfill the needs of tissues and maintain the homeostasis of the circulatory system. Endothelial phenotypic heterogeneity also plays an important role in the remodeling of the cardiovascular system where specific ECs are localized [3], [4].

The ductus arteriosus (DA), a fetal shunt artery between the pulmonary artery and the aorta, closes promptly after birth, although its connecting arteries remain open. The DA exhibits characteristics that are distinct from those of its connecting arteries. For example, the DA is more sensitive to the change in circulating oxygen concentration and prostaglandin E_2_ [5]–[7]. Since the changes in circulating oxygen concentration and prostaglandin E_2_ directly transduce the intravascular lumen where ECs surround its surface, ECs of the DA must play an important role in regulating these distinct characteristics. Accordingly, several studies have demonstrated the endothelium-dependent or independent vasoreaction of the DA [8]–[11]. Nonetheless, the majority of previous studies investigating molecular events in the DA utilized the whole DA tissue or cultured SMCs. Therefore, the role of DA ECs remains largely unknown. We hypothesized that the ECs of the DA exhibit a unique gene profile involved in DA-specific vasoconstriction and vascular remodeling. Recently, Weber et al. reported that they successfully isolated ECs from fetal rat DA using the immunomagnetic cell separation method and harvested the isolated ECs to further confirm their purity by flow cytometry analysis [12]. In the present study, we investigated gene expression differences in ECs between the DA and the aorta by using a combination of fluorescence-activated cell sorter (FACS) and DNA microarray experiments followed by further enrichment analysis using MetaCore GeneGo software.

## Materials and Methods

### Antibodies

FITC-conjugated anti-CD31 antibody was obtained from Abcam (Cambridge, MA, USA). APC/Cy7-conjugated anti-CD45, FITC-conjugated anti-control IgG, and APC/Cy7-conjugated anti-control IgG antibodies were obtained from Biolegend (San Diego, CA, USA).

### Animals

Timed-pregnant Wistar rats were purchased from Japan SLC, Inc. (Shizuoka, Japan). Rat fetuses at the 21st day of gestation as full-term were divided into two groups: fetuses before breathing (F group) and neonates obtained 30 minutes after breathing (N group). Animals in both groups were delivered by cesarean section. All animals were cared for in compliance with the American Physiological Society. The experiments were approved by the Ethical Committee on Animal Experiments of Waseda University.

### Fluorescence Activated Cell Sorter (FACS)

Pooled tissues from the DA or the aorta were obtained from three litters of timed-pregnant Wistar rats, which accounted for approximately thirty fetuses. Tissues were treated with collagenase-dispase enzyme mixture as described previously [13]. Approximately 1.0×10^6^ cells were obtained from combined whole DA tissues from the three litters. These cells were reacted with FITC-conjugated anti-CD31 and APC/Cy7-conjugated anti-CD45 antibodies as cell surface markers for EC and hematopoietic derivation cells, respectively. In order to confirm the nonspecific binding of antibodies to cells, we also prepared the cells reacted with a fluorescence conjugated anti-control IgG antibody. When dead cells reacted with PI solutions (Dojindo, Kumamoto, Japan), we found that about 30% of the cells (3.0×10^5^ cells) stained with PI solutions had died during isolation. The dead cells were then removed from further analysis. All cells were detected and sorted using BD FACSAria™II (Becton Dickinson, San Jose, CA, USA). In order to obtain as many cells as possible without affecting their purity, we set the FACS's sorting mode on “purity” in our cell sorter, which allowed the machine to automatically abort any possible forms of contamination, such as doublet cells, and to yield a purity of approximately more than 99%. In addition, we used the 100 µm size of the nozzle to decrease its sorting speed, which may contribute to an increase in accuracy. Therefore, we did not check the purity of the sorted endothelial cells by re-run, because the amount of ECs from rat DA was extremely small. The sorted cells were received by a 1.5 ml centrifuge tube containing 500 µl of DPBS (Wako, Osaka, Japan) and 0.4 µl of RNase inhibitor (Roche, Meylan, France).

### Quantitative real-time PCR (qRT-PCR)

The cell suspensions sorted by FACS were centrifuged at 300×g for 15 minutes, and the precipitation was quickly frozen in liquid nitrogen. Total RNA was extracted from the collected cells using an RNeasy micro kit (Qiagen, Valencia, CA, USA) according to the manufacturer's instructions. Total RNA was reverse-transcribed to cDNA using a High Capacity cDNA Reverse Transcription Kit (Applied Biosystems, Foster City, CA, USA). For quantitative RT-PCR analysis, sequences for PCR primers are listed in **Table 1**. qRT-PCR was performed using a Step One Real-time PCR System (Applied Biosystems) with Fast SYBR Green Master Mix (Applied Biosystems). The abundance of each gene was determined relative to an internal control using 18S rRNA. For each qRT-PCR experiment, which included an RT-negative control, we confirmed there was no non-specific amplification in any reaction.

**Table 1:** List of primer sequences used for quantitative RT-PCR.

Gene	NCBI	Primer sequence	
name*	Accession No.	Forward	Reverse
Tie2	NM_001105737.1	GGACAGTGCTCCAACCAAAT	CATCCCCAAAGTAAGGCTCA
γ2-actin	NM_012893.1	ATGTGGATCAGCAAGCCAGAG	GGTTTTAATGATCTGTGACTGGTGA
Ednra	NM_012550.2	CAACGGACCATCGCAGGAGCTTG	GGAGCCAGACGGAGCCTGAGC
Slc38a1	NM_138832.1	GTCCTGCCAATCTACAGCGA	GTACCCAAAGATGGCGGTCA
Lrat	NM_022280.2	CAGGCTGAGAAGTTTCACGA	CATCCACAAGCAGAACGGGA
18S	NR_003278.3	AGCCTGAGAAACGGCTACC	TCCCAAGATCCAACTACGAG
GAPDH	NM_017008.4	AGGTCGGTGTGAACGGATTTG	TGTAGACCATGTAGTTGAGGTCA

* The mRNA descriptions are listed below;

Tie2: TEK tyrosine kinase, endothelial; γ2-actin: actin, gamma 2, smooth muscle, enteric; Ednra: endothelin receptor type A; Slc38a1: solute carrier family 38, member 1; Lrat: lecithin-retinol acyltransferase; 18S: 18S ribosomal RNA; GAPDH: glyceraldehyde-3-phosphate dehydrogenase.

### DNA microarray procedure

We repeated FACS sorting ten times for each developmental group (30 litters used in total) in order to accumulate enough total RNA (∼100 ng). Then, cDNA was generated using the WT Expression Kit (Ambion, Austin, TX, USA) in accordance with the manufacturer's protocol. Briefly, a total of 100 ng of total RNA was reverse-transcribed to cDNA, which was subsequently used as a template for an *in vitro* transcription reaction. Sense-strand cDNA that contains dUTP was synthesized by amplified cRNA. We used the Affymetrix GeneChip® WT Terminal Labeling Kit (Affymetrix, Santa Clara, CA, USA) to recognize the dUTP and to fragment the cDNA with uracil-DNA glycosylase (UDG) and apurinic/apyrimidinic endonuclease 1 (APE1). These fragmented cDNAs were then labeled through a terminal deoxy-transferase reaction and hybridized to the Affymetrix GeneChip® Rat Gene 1.0 ST Array (Affymetrix). The hybridization experiments were performed in triplicate (approximately 180 litters were needed in total), and the intensities were averaged.

### Microarray data analysis

Of the 26,469 genes on the microarray, 14,944 were excluded based on aberrant low signals as determined by the poly-A spike of *lys* (probe set ID: 10700066) expression, the smallest composition out of the poly-A RNA control cocktail, which was added in each total RNA sample. All remaining gene probes were analyzed for their differential expression between the DA and the aorta at each developmental stage. Initially, we calculated the *p* value by Student's *t*-test across each group, and the data were cut off at *p*<0.05. For a more robust differential analysis between the DA and the aorta, we selected the genes that had more than a 2.0-fold change (|FC|≧2.0). Genes that went through these analyses were considered significant. Genes were further analyzed for enriched biological themes and pathways using the MetaCore program (GeneGo, a division of Thomson Reuters, St. Joseph, MI, USA). The program ranked the significant ontology and pathways dominant in DA ECs in each developmental stage by importing whole expression results (excluding aberrantly low expressed genes) from the microarray. MetaCore is an established program that includes a manually annotated database of gene interactions and metabolic reactions obtained from scientific literature. The enrichment analysis of the biological process was based on the hypergeometric distribution algorithm and relevant pathway maps were then prioritized according to their statistical significance [14]. The complete data set of the DNA microarray is available in the GEO database (accession number: GSE40500).

### Statistical treatment

Data are presented as mean ± standard error (SEM) or independent experiments. Statistical analyses were performed between two groups by unpaired two-tailed *t* test or unpaired *t* test with Welch correction, and among multiple groups by one-way analysis of variance (ANOVA) followed by Neuman-Keuls multiple comparison test. A *p* value of <0.05 was considered significant.

## Results

### Endothelial cells were purely isolated from rat DA tissues

**Figure 1:**
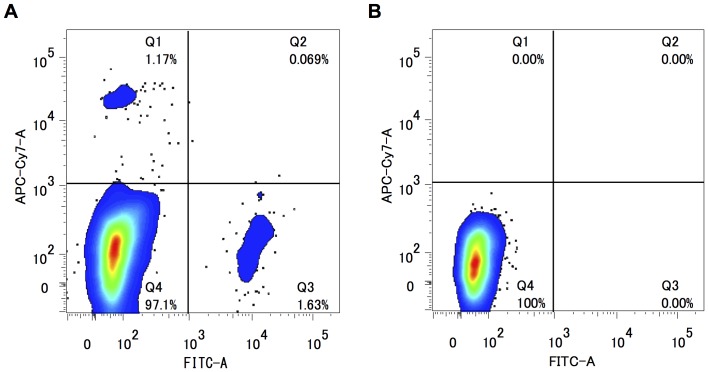
CD31+ cells successfully divided from whole tissue by FACS. A) Population of cells reacted with FITC-conjugated anti-CD31 antibody (CD31) and APC/Cy7-conjugated anti-CD45 antibody (CD45). CD31−/CD45−: consisting mainly of SMCs, CD31+/CD45−: consisting entirely of ECs B) Population of cells reacted with fluorescence conjugated anti-control IgG antibodies in order to confirm the nonspecific binding of antibodies.

**Figure 2:**
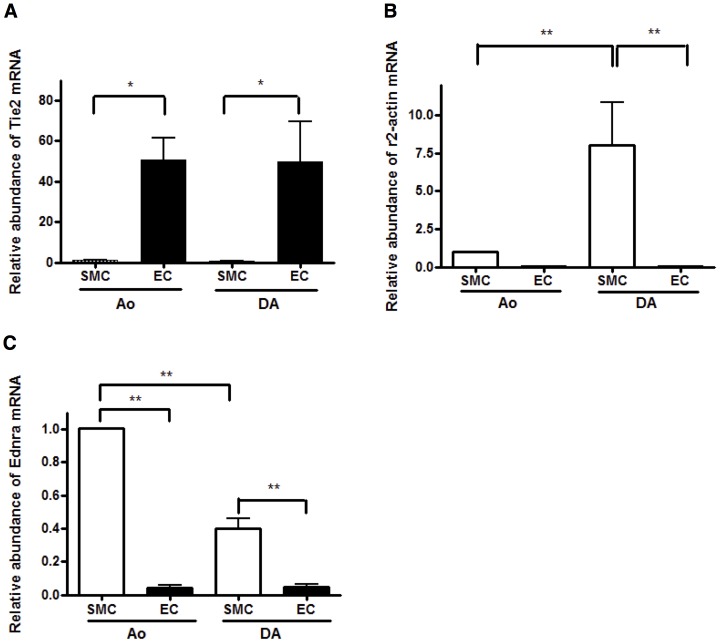
Obvious differences in gene expression between sorted ECs and SMCs. A) The expression levels of Tie2 mRNA were significantly higher in ECs than in SMCs. (*p<0.05, n = 5) B) The expression levels of γ2-actin mRNA were significantly lower in ECs than in SMCs. (**p<0.001, n = 5) C) The expression levels of Ednra mRNA were significantly lower in ECs than in SMCs. (**p<0.001, n = 3).

At least 10,000 of the cells (approximately 1% of the initially isolated cells) were sorted in anti-CD31 positive and anti-CD45 negative areas (CD31^+^/CD45^−^) from the pooled DA tissues of three litters of timed-pregnant Wistar rats (**Figure 1A**). No cell in the CD31^+^ area reacted with an anti-IgG antibody (**Figure 1B**), indicating that no false positive cells were contained in the CD31^+^/CD45^−^ cells believed to be ECs. We also assumed that CD31^−^/CD45^−^ cells mainly consisted of SMCs. The detailed gating strategies of FACS sorting are shown in **Figure S1**. To confirm the purity of FACS isolation, we examined the expression levels of EC-specific and SMC-specific genes by qRT-PCR. The expression levels of Tie2 mRNA, an EC-specific gene, were significantly higher in CD31^+^/CD45^−^ cells than in CD31^−^/CD45^−^ cells (*p*<0.05, n = 5) (**Figure 2A**). The expression levels of γ2-actin and endothelin-1 receptor Ednra mRNAs, SMC-specific genes, were significantly lower in CD31^+^/CD45^−^ cells than in CD31^−^/CD45^−^ cells (*p*<0.001, n = 3∼5) (**Figures 2B and 2C** **, respectively**). Therefore, we concluded that a FACS could isolate pure ECs in the CD31^+^/CD45^−^ area without contamination.

### Identification of DA-specific genes in ECs

**Figure 3:**
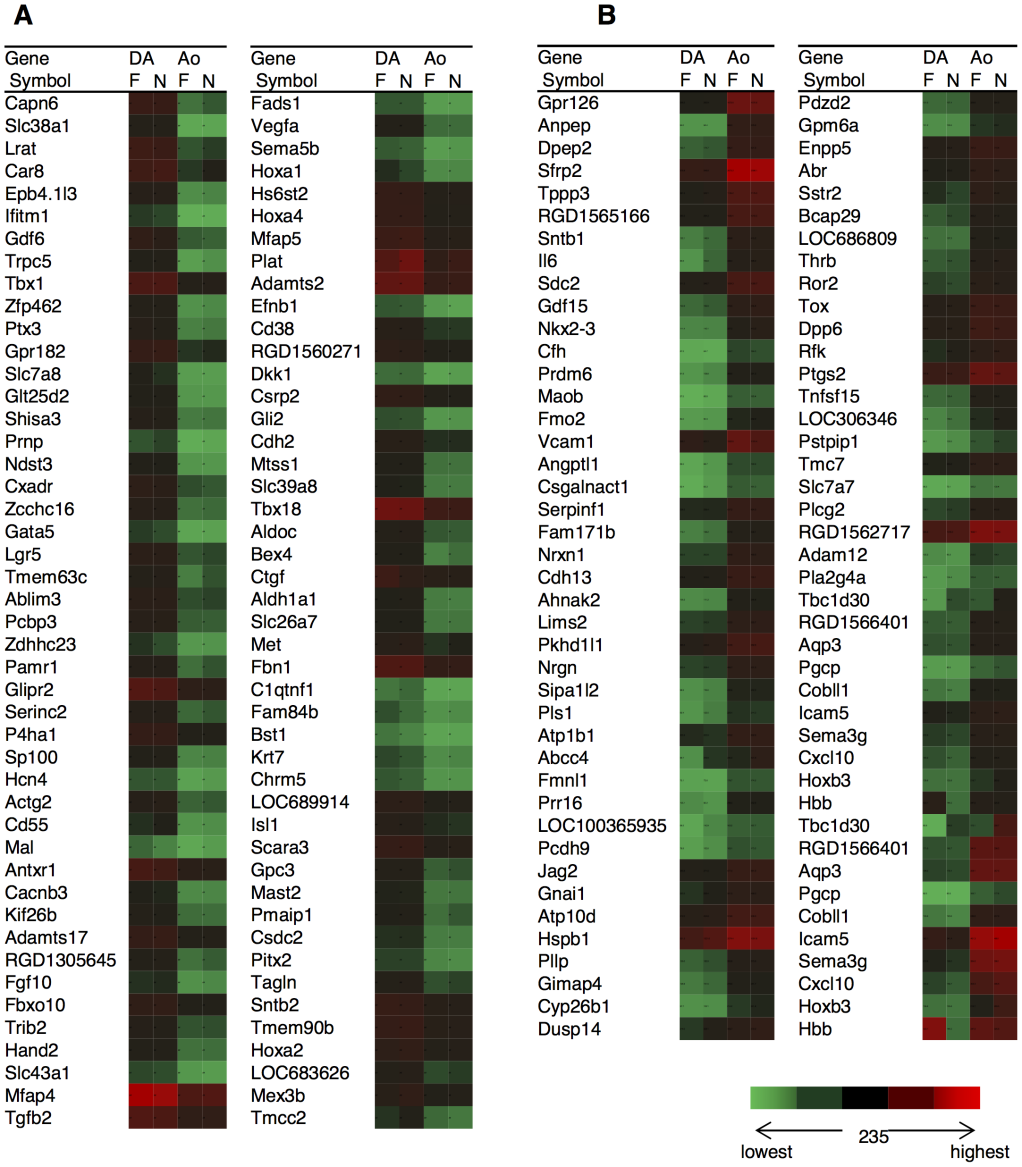
Color scale table imitating heat maps of DA dominant genes and Ao dominant genes. The listed genes in A) and B) are the same as in Table 2 and Table 3, respectively. The color scale is based on their expression intensities. The green or red color indicates the lowest or the highest expression levels, respectively. The midpoint shown as a dark color represents 235 since it is the average of whole gene expression.

Among over 26,469 gene-level probe sets, we found that 82 genes in the F group and 81 genes in the N group were expressed more than 2-fold in ECs of the DA than in ECs of the aorta (*p*<0.05) (**Table 2** **,** **Figure 3A**). Among these DA dominant genes, 71 genes were expressed more than 2-fold in ECs of the DA in both groups. On the other hand, 65 genes in the F group and 52 genes in the N group were expressed more than 2-fold in ECs of the aorta than in ECs of the DA (*p*<0.05) (**Table 3** **,** **Figure 3B**). Among these aorta dominant genes, 43 genes were expressed more than 2-fold in ECs of the aorta in both groups. Importantly, the majority of the genes in **Table 2** and **3** have never been reported previously as DA-related genes. We found only a limited number of the well-known endothelium-related genes such as transforming growth factor-beta 2 (Tgfb2) and vascular endothelial growth factor A (Vegfa). Validation of the results from the DNA microarray, qRT-PCR was performed with Slc38a1 and Lrat, the genes with the most significant difference between the DA and the aorta at both developmental stages (**Figure S2**).

**Table 2:** DA endothelium-dominant genes.

Probe	mRNA	Gene	Fold change (DA/Ao)	
set ID	Description	Symbol	F	N
10906592	solute carrier family 38, member 1	Slc38a1	7.31	6.81
10932759	calpain 6	Capn6	8.18	6.04
10823949	lecithin-retinol acyltransferase	Lrat	6.98	5.15
10875375	carbonic anhydrase 8	Car8	5.88	4.94
10925936	erythrocyte protein band 4.1-like 3	Epb4.1l3	5.05	4.27
10764702	similar to glycosyltransferase 25 domain containing 2	Glt25d2	3.90	4.18
10903177	G protein-coupled receptor 182	Gpr182	4.12	4.12
10712171	interferon induced transmembrane protein 1	Ifitm1	4.89	3.99
10867593	growth differentiation factor 6	Gdf6	4.82	3.89
10752295	T-box 1	Tbx1	4.69	3.80
10932726	transient receptor potential cation channel, subfamily C, member 5	Trpc5	4.73	3.78
10869158	similar to zinc finger protein 462	Zfp462	4.52	3.53
10840076	prion protein	Prnp	3.70	3.52
10826561	similar to N-deacetylase/N-sulfotransferase (heparan glucosaminyl) 3	Ndst3	3.69	3.36
10749983	coxsackie virus and adenovirus receptor	Cxadr	3.65	3.36
10937327	similar to zinc finger, CCHC domain containing 5	Zcchc16	3.63	3.32
10783648	solute carrier family 7, member 8	Slc7a8	3.95	3.25
10776676	shisa homolog 3 (Xenopus laevis)	Shisa3	3.76	3.16
10815785	pentraxin related gene	Ptx3	4.28	3.14
10829418	poly(rC) binding protein 3	Pcbp3	3.01	3.10
10714323	aldehyde dehydrogenase 1 family, member A1	Aldh1a1	2.19	3.08
10924824	SP100 nuclear antigen	Sp100	2.83	3.00
10863549	actin, gamma 2, smooth muscle, enteric	Actg2	2.76	2.96
10852378	GATA binding protein 5	Gata5	3.60	2.91
10931308	prolyl 4-hydroxylase, alpha polypeptide I	P4ha1	2.86	2.89
10804750	similar to Actin-binding LIM protein 3	Ablim3	3.15	2.88
10902420	leucine rich repeat containing G protein coupled receptor 5	Lgr5	3.49	2.84
10862554	31 kDa protein	Hoxa4	2.42	2.77
10767388	Cd55 molecule	Cd55	2.75	2.76
10868627	similar to GLI pathogenesis-related 2	Glipr2	2.87	2.75
10934173	ephrin B1	Efnb1	2.35	2.66
10876507	similar to F-box only protein 10	Fbxo10	2.56	2.65
10935038	brain expressed gene 4	Bex4	2.2	2.62
10837351	solute carrier family 43, member 1	Slc43a1	2.52	2.60
10707862	similar to ADAM metallopeptidase with thrombospondin type 1 motif, 17 preproprotein	Adamts17	2.61	2.60
10880095	serine incorporator 2	Serinc2	2.87	2.56
10838117	peptidase domain containing associated with muscle regeneration 1	Pamr1	2.95	2.55
10886162	transmembrane protein 63c	Tmem63c	3.49	2.53
10745095	aldolase C, fructose-bisphosphate	Aldoc	2.22	2.51
10833152	cysteine and glycine-rich protein 2	Csrp2	2.31	2.48
10739927	C1q and tumor necrosis factor related protein 1	C1qtnf1	2.15	2.48
10858499	microfibrillar associated protein 5	Mfap5	2.42	2.46
10792421	plasminogen activator, tissue	Plat	2.41	2.46
10853819	met proto-oncogene	Met	2.17	2.44
10791504	heart and neural crest derivatives expressed 2	Hand2	2.52	2.42
10713857	fatty acid desaturase 1	Fads1	2.46	2.38
10899023	calcium channel, voltage-dependent, beta 3 subunit	Cacnb3	2.67	2.38
10863777	anthrax toxin receptor 1	Antxr1	2.70	2.37
10813172	fibroblast growth factor 10	Fgf10	2.58	2.37
10939764	glypican 3	Gpc3	2.04	2.36
10766082	kinesin family member 26B	Kif26b	2.65	2.35
10896751	metastasis suppressor 1	Mtss1	2.27	2.35
10921772	vascular endothelial growth factor A, transcript variant 1	Vegfa	2.45	2.34
10733258	ADAM metallopeptidase with thrombospondin type 1 motif, 2	Adamts2	2.40	2.30
10910473	hyperpolarization activated cyclic nucleotide-gated potassium channel 4	Hcn4	2.81	2.29
10751190	zinc finger, DHHC-type containing 23	Zdhhc23	2.96	2.24
10777232	CD38 molecule	Cd38	2.34	2.21
10919175	T-box18	Tbx18	2.23	2.21
10803323	cadherin 2	Cdh2	2.28	2.20
10939725	similar to Heparan-sulfate 6-O-sulfotransferase 2	Hs6st2	2.44	2.19
10922964	similar to esophageal cancer related gene 4 protein	RGD1305645	2.60	2.17
10770577	transforming growth factor, beta 2	Tgfb2	2.50	2.17
10875581	solute carrier family 26, member 7	Slc26a7	2.18	2.17
10734242	microfibrillar-associated protein 4	Mfap4	2.51	2.15
10889263	tribbles homolog 2 (Drosophila)	Trib2	2.55	2.11
10767077	GLI family zinc finger 2	Gli2	2.29	2.11
10921428	similar to inhibitor of MyoD family-a	RGD1560271	2.34	2.10
10819269	solute carrier family 39 (zinc transporter), member 8	Slc39a8	2.23	2.08
10784579	scavenger receptor class A, member 3	Scara3	2.04	2.05
10849327	fibrillin 1	Fbn1	2.15	2.04
10729667	dickkopf homolog 1 (Xenopus laevis)	Dkk1	2.32	2.02
10767597	similar to transmembrane and coiled-coil domains 2	Tmcc2	1.54	2.48
10888610	similar to limb-bud and heart	LOC683626	1.71	2.24
10818989	paired-like homeodomain 2, transcript variant 2	Pitx2	1.96	2.14
10917034	transgelin	Tagln	1.95	2.13
10708399	similar to ring finger and KH domain containing 3	Mex3b	1.68	2.12
10840613	transmembrane protein 90B	Tmem90b	1.85	2.11
10807601	syntrophin, beta 2	Sntb2	1.93	2.02
10862547	homeo box A2	Hoxa2	1.76	2.01
10898022	cold shock domain containing C2, RNA binding	Csdc2	1.97	2.00
10802375	phorbol-12-myristate-13-acetate-induced protein 1	Pmaip1	1.99	2.00
10849700	mal, T-cell differentiation protein	Mal	2.71	1.51
10754454	semaphorin 5B	Sema5b	2.44	1.93
10862541	homeo box A1	Hoxa1	2.44	1.95
10717233	connective tissue growth factor	Ctgf	2.19	1.29
10903979	similar to breast cancer membrane protein 101 isoform 1	Fam84b	2.15	1.51
10777242	bone marrow stromal cell antigen 1	Bst1	2.12	1.68
10899405	Keratin, type II cytoskeletal 7	Krt7	2.09	1.66
10848165	cholinergic receptor, muscarinic 5	Chrm5	2.09	1.89
10871043	similar to C05G5.5	LOC689914	2.07	1.89
10821486	ISL LIM homeobox 1	Isl1	2.05	1.93
10878845	microtubule associated serine/threonine kinase 2	Mast2	2.02	1.93

Eighty two genes in the F group and 81 genes in the N group were expressed more than 2-fold in ECs of the DA than in ECs of the aorta (*p*<0.05). Among these DA dominant genes, 71 genes were expressed more than 2-fold in ECs of the DA in both groups (above the thick line). F: fetuses before breathing; N: neonates obtained 30 minutes after breathing.

**Table 3:** Aorta endothelium-dominant genes.

Probe	mRNA	Gene	Fold change (DA/Ao)	
set ID	Description	Symbol	F	N
10722992	alanyl (membrane) aminopeptidase	Anpep	0.16	0.16
10716939	similar to G protein-coupled receptor 126	Gpr126	0.15	0.19
10816144	secreted frizzled-related protein 2	Sfrp2	0.25	0.25
10810778	dipeptidase 2	Dpep2	0.25	0.28
10730266	NK2 transcription factor related, locus 3 (Drosophila)	Nkx2–3	0.33	0.28
10731622	similar to MGC45438 protein	RGD1565166	0.26	0.28
10810631	tubulin polymerization-promoting protein family member 3	Tppp3	0.26	0.29
10787517	growth differentiation factor 15	Gdf15	0.32	0.31
10896020	syndecan 2	Sdc2	0.31	0.33
10769370	flavin containing monooxygenase 2	Fmo2	0.35	0.34
10826249	vascular cell adhesion molecule 1	Vcam1	0.35	0.35
10768269	complement factor H	Cfh	0.34	0.35
10903816	syntrophin, beta 1	Sntb1	0.27	0.36
10801683	proline rich 16	Prr16	0.42	0.36
10769476	ATPase, Na+/K+ transporting, beta 1 polypeptide	Atp1b1	0.41	0.37
10801761	similar to PR-domain zinc finger protein 6	Prdm6	0.34	0.37
10744939	serine (or cysteine) peptidase inhibitor, clade F, member 1	Serpinf1	0.37	0.38
10859799	interleukin 6	Il6	0.27	0.39
10892352	similar to Jagged-2 precursor	Jag2	0.44	0.40
10800696	LIM and senescent cell antigen like domains 2	Lims2	0.40	0.41
10888368	neurexin 1	Nrxn1	0.37	0.41
10916228	neurogranin	Nrgn	0.40	0.42
10932211	monoamine oxidase B, nuclear gene encoding mitochondrial protein	Maob	0.35	0.42
10808274	cadherin 13	Cdh13	0.38	0.42
10738676	formin-like 1	Fmnl1	0.42	0.42
10863608	cytochrome P450, family 26, subfamily b, polypeptide 1	Cyp26b1	0.46	0.42
10785846	ATP-binding cassette, sub-family C (CFTR/MRP), member 4	Abcc4	0.41	0.44
10892330	similar to AHNAK nucleoprotein isoform 1	Ahnak2	0.38	0.45
10837310	similar to KIAA1946	Fam171b	0.37	0.45
10896405	polycystic kidney and hepatic disease 1-like 1	Pkhd1l1	0.40	0.45
10791552	glycoprotein m6a	Gpm6a	0.46	0.45
10764862	angiopoietin-like 1	Angptl1	0.37	0.46
10782454	thyroid hormone receptor beta	Thrb	0.48	0.46
10822007	PDZ domain containing 2	Pdzd2	0.46	0.47
10726371	similar to ADAM 12 precursor	Adam12	0.50	0.48
10811956	signal-induced proliferation-associated 1 like 2	Sipa1l2	0.41	0.48
10739364	somatostatin receptor 2	Sstr2	0.47	0.48
10790939	similar to KIAA1683	LOC306346	0.49	0.48
10797499	receptor tyrosine kinase-like orphan receptor 2	Ror2	0.48	0.49
10704840	similar to protein 7 transactivated by hepatitis B virus X antigen	LOC686809	0.47	0.49
10805996	plasma membrane proteolipid (plasmolipin)	Pllp	0.45	0.49
10776608	similar to Probable phospholipid-transporting ATPase VD	Atp10d	0.45	0.5
10859886	dipeptidylpeptidase 6	Dpp6	0.48	0.50
10724315	hemoglobin, beta	Hbb	1.21	0.44
10896028	plasma glutamate carboxypeptidase	Pgcp	0.53	0.47
10771655	chemokine (C-X-C motif) ligand 10	Cxcl10	0.61	0.48
10737730	homeo box B3	Hoxb3	0.64	0.48
10845767	Cobl-like 1	Cobll1	0.54	0.49
10886816	cDNA clone IMAGE:8372043.	RGD1566401	0.5	0.49
10908328	intercellular adhesion molecule 5, telencephalin	Icam5	0.56	0.49
10876069	aquaporin 3	Aqp3	0.51	0.50
10786646	semaphorin 3G	Sema3g	0.56	0.50
10736520	active BCR-related gene	Abr	0.46	0.54
10889560	B-cell receptor-associated protein 29	Bcap29	0.47	0.63
10787757	chondroitin sulfate N-acetylgalactosaminyltransferase 1	Csgalnact1	0.37	0.52
10834031	dual specificity phosphatase 14	Dusp14	0.46	0.54
10926651	ectonucleotide pyrophosphatase/phosphodiesterase 5	Enpp5	0.46	0.52
10855387	GTPase, IMAP family member 4	Gimap4	0.45	0.58
10853229	guanine nucleotide binding protein (G protein), alpha inhibiting 1	Gnai1	0.45	0.55
10761128	heat shock protein 1	Hspb1	0.45	0.62
10833346	cDNA clone MGC:188337 IMAGE:7453022	LOC100365935	0.42	0.66
10785523	similar to Protocadherin 9 precursor isoform 3	Pcdh9	0.43	0.58
10768376	phospholipase A2, group IVA (cytosolic, calcium-dependent)	Pla2g4a	0.50	0.63
10811347	phospholipase C, gamma 2	Plcg2	0.49	0.51
10919354	plastin 1 (I isoform)	Pls1	0.41	0.57
10910204	proline-serine-threonine phosphatase-interacting protein 1	Pstpip1	0.49	0.51
10764551	prostaglandin-endoperoxide synthase 2	Ptgs2	0.48	0.52
10934662	riboflavin kinase	Rfk	0.48	0.56
10753629	126 kDa protein	RGD1562717	0.49	0.55
10783537	solute carrier family 7, member 7	Slc7a7	0.49	0.56
10902696	similar to CG3996-PA	Tbc1d30	0.50	0.63
10725253	similar to Tmc7 protein	Tmc7	0.49	0.51
10877532	tumor necrosis factor (ligand) superfamily, member 15	Tnfsf15	0.48	0.54
10875363	thymocyte selection-associated high mobility group box	Tox	0.48	0.51

Sixty five genes in the F group and 52 genes in the N group were expressed more than 2-fold in ECs of the aorta than in ECs of the DA (*p*<0.05). Among these aorta dominant genes, 43 genes were expressed more than 2-fold in ECs of the aorta in both groups(above the thick line). F: fetuses before breathing; N: neonates obtained 30 minutes after breathing.

Surprisingly, the present results showed remarkably low variations of transcription profiles before and after birth. Although there were 178 genes of which expression levels significantly differed between both developmental stages (*p*<0.05), arrestin domain containing 3 (Arrdc3) and TBC1 domain family, member 30 (Tbc1d30) were the only two genes that had more than a 2.0-fold change (|FC|≧2.0) between both developmental stages. Among 178 genes, **Table 4** shows 25 genes of which the *p* values were less than 0.01 (*p*<0.01). Among these 25 genes, connective tissue growth factor (Ctgf) and Tbc1d30 are listed in **Table 2** (DA-dominant) and **Table 3** (aorta-dominant), respectively.

**Table 4:** Genes showed a significant change (*p*<0.01) between F and N in the DA.

Probe set ID	mRNA Description	Gene Symbol	*p*-value (F vs N)
10869253	UDP-glucose ceramide glucosyltransferase	Ugcg	0.0005
10728164	protein phosphatase 2, regulatory subunit B', beta isoform	Ppp2r5b	0.0012
10767489	mitogen-activated protein kinase-activated protein kinase 2	Mapkapk2	0.0015
10902696	similar to CG3996-PA	Tbc1d30	0.0015
10927612	similar to TBC1 domain family, member 8 isoform 3	Tbc1d8	0.0023
10912584	angiomotin like 2	Amotl2	0.0026
10825940	adenosine monophosphate deaminase 2 (isoform L)	Ampd2	0.0028
10940568	dicarbonyl L-xylulose reductase	Dcxr	0.0028
10861066	transmembrane protein 106B	Tmem106b	0.0030
10885235	protein kinase C, eta	Prkch	0.0044
10879257	cell division cycle 20 homolog (S. cerevisiae)	Cdc20	0.0054
10895310	protein phosphatase 1, regulatory (inhibitor) subunit 12A	Ppp1r12a	0.0057
10863542	deoxyguanosine kinase, nuclear gene encoding mitochondrial protein	Dguok	0.0057
10717233	connective tissue growth factor	Ctgf	0.0063
10727464	ribosomal protein S6 kinase, polypeptide 2	Rps6kb2	0.0074
10836973	secernin 3	Scrn3	0.0075
10857655	LIM and cysteine-rich domains 1	Lmcd1	0.0079
10773088	LIM domain binding 2	Ldb2	0.0080
10832197	SNF1-like kinase	Sik1	0.0085
10864715	vestigial like 4 (Drosophila)	Vgll4	0.0087
10826371	palmdelphin	Palmd	0.0088
10730792	hypothetical protein	Itprip	0.0090
10901713	nicolin 1	Nicn1	0.0097
10884898	similar to Voltage-dependent anion-selective channel protein 1	RGD1565338	0.0098
10817309	phosphatidylinositol 4-kinase, catalytic, beta	Pi4kb	0.0100

Twenty five genes shows a significant difference between F and N groups in the DA (*p* values were less than 0.01. Among these 25 genes, Ctgf and Tbc1d30 are listed in Table 2 and Table 3, respectively. F: fetuses before breathing; N: neonates obtained 30 minutes after breathing.

### Enrichment analysis of DA dominant genes using GeneGo MetaCore software

**Figure 4:**
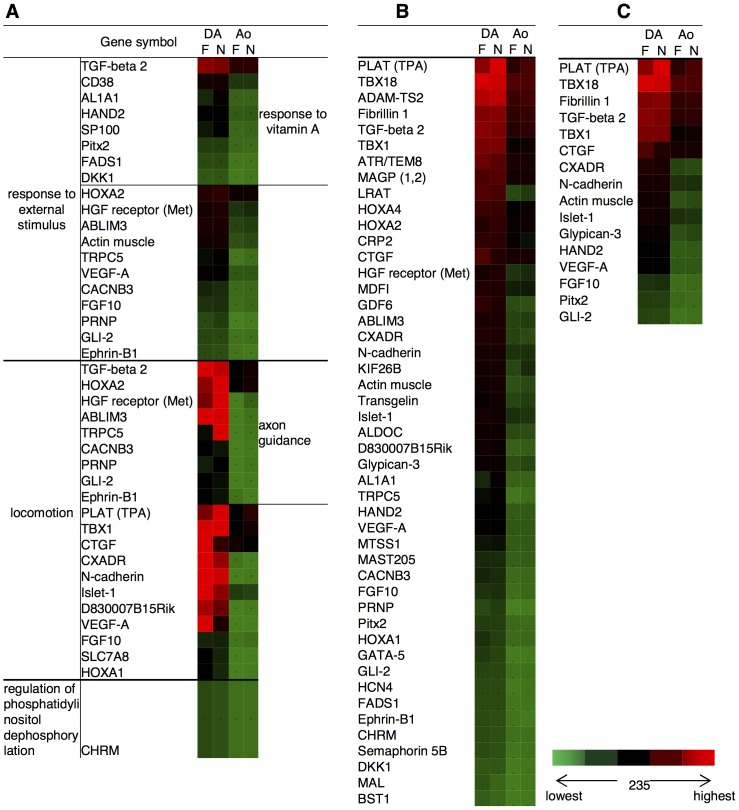
Color scale table imitating heat maps of the DA dominant genes categorized by GeneGo processes. DA dominant genes are identified using GO analysis (MetaCore). The whole expression data set was processed by importing it into the MetaCore system. The MetaCore system lined up the top 10 (based on *p*-value) sets of categorized genes according to their GO biological processes (Table 5). The color scale table imitating heat maps was created manually based on the genes in GO biological processes. A) The genes in all the top processes except the development or morphogenesis processes that emerged in both F and N. B) The genes categorized in the processes related to the development and morphogenesis in both F and N. C) The genes categorized only in cardiovascular or circulatory specific development processes. The color scale is the same as that used in Figure 3. All heat maps were created manually based on the genes in the GeneGo biological processes. a) The genes in all the processes in Table 4, except the development or morphogenesis processes which emerged in both F and N. b) The genes categorized in the processes related to development and morphogenesis in both F and N. c) The genes categorized only in cardiovascular or circulatory specific development processes.

In the MetaCore systems, there are about 110 cellular and molecular processes whose content is defined and annotated by GeneGo. The top 10 ranked regulatory biological processes were listed in each stage of the DA ECs based upon their *p* values (**Table 5**). Most of the categories indicate morphogenesis and development. Four processes (anatomical structure morphogenesis, cardiovascular system development, circulatory system development, and locomotion) are ranked in both the F and N groups. Interestingly, excluding processes related to morphogenesis and development, regulation of phosphatidylinositol dephosphorylation is an enriched process that is listed only in the F group. On the other hand, response to external stimulus, response to vitamin A stimulus, and axon guidance were listed only in the top 10 ranked biological processes in the N group. In these GeneGo biological processes, 322 and 172 genes were listed in the F and N groups, respectively. The genes included in each category are shown in **Figure 4**. There are a considerable number of overlapping genes in each process. **Table 6** shows the 30 genes that frequently appeared in more than five processes of the top 10 ranking as active genes. These genes are likely to be involved in the network by potential interactions with many of the identified genes to form DA-specific endothelium.

**Table 5:** Top 10 regulatory biological processes worked in the DA ECs.

GO Processes	*p*-value (DA vs Ao)	Developmental stage
Anatomical structure morphogenesis	8.91E-13	F
Circulatory system development	2.41E-12	F
Cardiovascular system development	2.41E-12	F
Developmental process	4.04E-12	F
Locomotion	6.97E-12	F
Multicellular organismal development	4.41E-11	F
Organ morphogenesis	1.55E-10	F
System development	1.56E-10	F
Anatomical structure development	2.86E-10	F
Regulation of phosphatidylinositol dephosphorylation	3.07E-10	F
Muscle structure development	1.35E-10	N
Anatomical structure morphogenesis	4.64E-10	N
Response to external stimulus	3.98E-09	N
Muscle cell differentiation	4.00E-09	N
Locomotion	1.68E-08	N
Circulatory system development	1.70E-08	N
Cardiovascular system development	1.70E-08	N
Tissue morphogenesis	2.81E-08	N
Axon guidance	3.47E-08	N
Response to vitamin A	3.50E-08	N

The MetaCore systems defined the top 10 ranked regulatory biological processes that were dominantly worked in each stage of the DA ECs based upon their *p* values. F: fetuses before breathing; N: neonates obtained 30 minutes after breathing.

**Table 6:** Thirty overlapping genes that appeared in more than five processes of the top ten ranking as active genes.

ID	Gene Symbol	mRNA- Description	Number of overlapped processes	
			F	N
***Receptor ligand***				
10770577	Tgfb2	transforming growth factor, beta 2 (Tgfb2)	9	9
10921772	Vegfa	vascular endothelial growth factor A (Vegfa), transcript variant 1	9	8
10813172	Fgf10	fibroblast growth factor 10 (Fgf10)	9	8
10934173	Efnb1	ephrin B1 (Efnb1)	9	6
10717233	Ctgf	connective tissue growth factor (Ctgf)	9	0
10849327	Fbn1	fibrillin 1 (Fbn1)	8	3
***Receptor***				
10749983	Cxadr	coxsackie virus and adenovirus receptor (Cxadr)	7	5
10853819	Met	met proto-oncogene (Met)	6	6
10848165	Chrm5	cholinergic receptor, muscarinic 5 (Chrm5)	5	0
***Voltage-gated ion channel***				
10899023	Cacnb3	calcium channel, voltage-dependent, beta 3 subunit (Cacnb3)	6	6
10932726	Trpc5	transient receptor potential cation channel, subfamily C, member 5 (Trpc5)	6	4
***Binding protein***				
10863549	Actg2	actin, gamma 2, smooth muscle, enteric (Actg2)	9	9
10803323	Cdh2	cadherin 2 (Cdh2)	8	6
10939764	Gpc3	glypican 3 (Gpc3)	8	0
10804750	Ablim3	actin-binding LIM protein 3 gene:ENSRNOG00000019365	6	4
10840076	Prnp	prion protein (Prnp)	6	4
10766072	Kif26b	kinesin family member 26B (Kif26b)	6	2
10921428	RGD1560271	similar to inhibitor of MyoD family-a	6	1
10858499	Mfap5	microfibrillar associated protein 5 (Mfap5)	5	1
***Transcriptional factor***				
10767077	Gli2	GLI family zinc finger 2 (Gli2)	9	7
10752295	Tbx1	T-box 1 (Tbx1)	9	7
10821486	Isl1	ISL LIM homeobox 1 (Isl1)	9	0
10791504	Hand2	heart and neural crest derivatives expressed 2 (Hand2)	8	6
10919175	Tbx18	T-box18 (Tbx18)	7	6
10862541	Hoxa1	homeo box A1 (Hoxa1)	7	0
10862554	Hoxa4	homeo box A4 (Hoxa4)	6	0
10818989	Pitx2	paired-like homeodomain 2 (Pitx2), transcript variant 2	0	8
***enzyme***				
10792421	Plat	plasminogen activator, tissue (Plat)	8	4
10714323	Aldh1a1	aldehyde dehydrogenase 1 family, member A1 (Aldh1a1)	6	4
10745095	Aldoc	aldolase C, fructose-bisphosphate (Aldoc)	6	1

Thirty genes that frequently appeared in more than five processes of the top ten ranking in Table 5 are regarded as active genes. These genes are listed in accordance with their function. F: fetuses before breathing; N: neonates obtained 30 minutes after breathing.

Furthermore, there are over 1200 pathway maps in MetaCore, comprehensively covering signaling and metabolism, selected diseases and some drug targets mechanisms. All maps are accurately drawn by GeneGo annotators and manually curated and edited. The canonical pathway maps and GeneGo process networks, validated by statistical values, were evaluated by MetaCore and are listed in **Table 7** together with the top 10 ranking for each pathway significantly worked in the DA ECs. As we found that the gene expression profiles exhibited remarkably low variations at both time points, nine of the top 10 ranked pathway maps were listed in both F and N groups. These categories are related to regulation of epithelial-to-mesenchymal transition (EMT), cell adhesion, and retinol metabolism.

**Table 7:** Top 10 pathways arranged by *p*-value.

GeneGo Pathway Maps	*p*-value (DA vs Ao)	Developmental stage
Development_Regulation of epithelial-to-mesenchymal transition (EMT)	7.52E-05	F
Cell adhesion_Cadherin-mediated cell adhesion	1.04E-04	F
Cell adhesion_Plasmin signaling	2.56E-04	F
Development_TGF-beta-dependent induction of EMT via SMADs	2.56E-04	F
Development_TGF-beta-dependent induction of EMT via RhoA, PI3K and ILK.	5.79E-04	F
Retinol metabolism/Rodent version	1.97E-03	F
Retinol metabolism	2.31E-03	F
Development_S1P2 and S1P3 receptors in cell proliferation and differentiation	3.87E-03	F
Cell adhesion_Chemokines and adhesion	5.41E-03	F
Cytoskeleton remodeling_TGF, WNT and cytoskeletal remodeling	7.23E-03	F
Development_Regulation of epithelial-to-mesenchymal transition (EMT)	7.52E-05	N
Cell adhesion_Cadherin-mediated cell adhesion	1.04E-04	N
Development_S1P2 and S1P3 receptors in cell proliferation and differentiation	1.04E-04	N
Cell adhesion_Plasmin signaling	2.56E-04	N
Development_TGF-beta-dependent induction of EMT via SMADs	2.56E-04	N
Development_TGF-beta-dependent induction of EMT via RhoA, PI3K and ILK.	5.79E-04	N
Retinol metabolism/Rodent version	1.97E-03	N
Retinol metabolism	2.31E-03	N
Muscle contraction_nNOS Signaling in Skeletal Muscle	4.49E-03	N
Cell adhesion_Chemokines and adhesion	5.41E-03	N

Among over 1200 pathways, the MetaCore systems defined the top 10 ranked pathways that were dominantly worked in each stage of the DA ECs based upon their *p* values. F: fetuses before breathing; N: neonates obtained 30 minutes after breathing.

## Discussion

To date, the characteristic features of the DA endothelium have remained largely unknown. Several studies have demonstrated the endothelium-dependent or independent vasomotor reaction of the DA [8]–[11]. Rabinovitch et al. made great efforts to identify the role of the DA endothelium in vascular remodeling of the DA. They found that the increase in the expression of transforming growth factor-beta (Tgfb) in the DA endothelium promoted the synthesis of glycosaminoglycan such as hyaluronan that is a critical regulator of neointimal formation of the DA [15], [16]. The present comprehensive gene expression analysis identified a DA endothelium-dominant rat gene profile during a perinatal period for the first time. It should be noted that we collected DA ECs from more than 30 litters to obtain a sufficient amount of mRNA for one sample. Additionally, the hybridization experiments were performed in triplicate and the intensities were averaged. To avoid an unexpected artificial bias, we did not use cultured ECs or any amplification method to increase mRNA from the endothelium. Therefore, the present study represents the transcription profile of the freshly isolated endothelium from the rat DA. Importantly, most of the genes that were expressed greater or lower in the DA endothelium than in the aortic endothelium have not yet been investigated in the DA. In addition to the up-regulated or down-regulated genes that met the 2-fold threshold in **Table 2** and **3**, one may be interested in genes that showed a statistical significance but a lower than 2.0 difference. We therefore also listed the genes with a statistical significance (*p*<0.001) in **Table S1 and Table S2**. Since the endothelium plays a critical role not only in vascular tone but also in vascular remodeling, the newly identified genes should be of great interest for further investigation of the molecular mechanisms of DA-specific differentiation and function.

Although two studies, including our previous one, have identified DA-dominant genes using DNA microarray analysis [17], [18], the present study revealed that the transcription profile of DA ECs are quite different from those of DA whole tissues of which the majority is composed of SMCs. These data suggest that the transcription profiles of the DA endothelium are tightly regulated in a cell-specific manner. Furthermore, local interaction between ECs and SMCs may contribute to establishing each unique transcription profile in the DA. It would be beneficial to further investigate how ECs and SMCs interact locally with each other.

To our surprise, the present study also demonstrated that the transcription profile of DA ECs did not change much before and after birth, although the DA does dramatically alter its morphology during the perinatal period. After birth, the change in oxygen and PGE_2_ content in circulating blood induces functional closure of the DA. Costa et al. also demonstrated that the transcription profile of DA tissues significantly differs before and after birth [18]. In their experiment, DA samples were collected 3 hours after spontaneous delivery. We used neonatal DA ECs 30 minutes after delivery by cesarean section, because we aimed to detect an initial change in the transcription profile of DA ECs after birth. This period, however, may not be long enough to investigate the alternation in its transcription profile, although functional closure of the rat DA had mostly occurred in our previous studies [19], [20].

It is very important to investigate the roles of the newly identified genes in the morphology and function of the DA. Unfortunately, an *in vitro* experiment using rat DA ECs is technically very difficult because of the limited amount of tissue or cells obtainable from small animals. Currently, bioinformatic technology has developed to the point that it is now possible to attribute functions to genes and their encoded proteins, and to identify the regulatory networks controlling metabolic, protein synthesis and signal transduction pathways. To facilitate the analysis of experiments using post-genomic technologies, newly developed knowledge-based gene set enrichment analysis provides a powerful analytical method to link the vast amount of raw data to biological pathways [14], [21]. Pathway analysis by MetaCore is based on the concept that the function of a gene depends directly on the context in which it acts, and MetaCore correlates genes identified by DNA microarray with the cellular pathways that are hypothetically activated dominantly in the DA endothelium. The present study revealed possible regulatory factors involved in specific “process networks” such as the regulation of morphogenesis and development. We found a considerable number of overlapping genes in these processes that are likely to be involved in the network through potential interactions with many of the identified genes. The genes listed in **Table 6** are considered the “functional hubs” of the DA endothelium-specific network.

Eight transcription factors in **Table 6** that have been known to be involved in formation of the cardiovascular system may play an important role in forming the endothelial phenotypic heterogeneity of the DA, although none of them has been intensively investigated in the DA. Although several previous studies have demonstrated that the transcription factors Tfap2β, Hif2α, and myocardin play an important role in ductal smooth muscle development [22], [23], the transcriptional regulation of DA endothelium-specific differentiation remains largely unknown. The DA originates from the sixth pharyngeal arch artery that initially forms as protuberances of the dorsal aorta and the aortic sac [24]. The cells that comprise the pharyngeal arch arteries are of pharyngeal mesoderm origin. The mesodermal core of the arches is continuous with the mesoderm derived from the second heart field (SHF) [25]. To date, the majority of the cells that constitute the DA media are known to derive from cardiac neural crest cells (NCCs) at the somite 1 to somite 3 level [26], [27]. The importance of this neural crest origin in understanding specific DA differentiation lies in the segmental nature of the pharyngeal arches themselves and of the origin of the NCCs that invade them. Accordingly, transcription factors related to NCCs such as Hoxa1, Hoxa4, and heart and neural crest derivatives expressed 2 (Hand2) [28]–[30] are listed in **Table 6**. Although a previous study suggested that Hoxb5 may be involved in DA differentiation [31], the expression level of Hoxb5 mRNA was not increased in the DA ECs in the present study. A recent study in humans revealed that mutations in Hoxa1 can cause severe cardiovascular malformations in patients with Bosley-Salih-Alorainy Syndrome [32]. Furthermore, Hoxa1 null mice show defects such as interrupted aortic arch, aberrant subclavian artery and tetralogy of Fallot, demonstrating that Hoxa1 is required for patterning of the great arteries and outflow tract of the heart [28].

In addition, MetaCore enrichment analysis revealed that the SHF-related transcription factors T-box (Tbx) 1, Tbx18, and Isl1, and the receptor ligand Fgf10 are enriched in the DA endothelium. It has also been shown that Isl1, Fgf10-positive mesoderm of the posterior arches forms the ECs of arterial blood vessels^25^. Although Rana et al. demonstrated that the endothelium of the pharyngeal arch arteries is largely negative for Tbx1 [33], its expression levels were greater in the DA ECs than in the aortic ECs in the present study. Therefore, the results indicated that the SHF-derived cells are more prevalent in the DA endothelium than in the descending aortic endothelium. To our knowledge, no study has reported that the SHF-derived cells contribute to DA differentiation. Isl1 and Tbx1 regulate Fgf10 transcription in the SHF during cardiac outflow formation [34], [35]. It would be of value to further investigate the interaction between the NCC-derived and SHF-derived cells in the DA.

Furthermore, MetaCore enrichment analysis identified that the epithelial-to-mesenchymal transition (EMT) pathway functional in the DA ECs. The key genes Tgfb2, actin, alpha 2, smooth muscle, aorta (ACTA2), N-cadherin (cadherin 2, Cdh2), and met proto-oncogene (hepatocyte growth factor receptor, Met) are listed in the pathway. One of the characteristics of the DA vascular remodeling is physiological intimal thickness that is profound during the perinatal period. Within certain environments, endothelial-to-mesenchymal transition (EnMT) plays a role in promoting arterial intimal hyperplasia [36], [37]. EnMT shares a number of molecular signaling pathways with Tgfb1-induced EMT [38], [39], although it has not been proven that migrated ECs of the DA into the subendothelium differentiate into smooth muscle-like (mesenchymal) cells. Gittenberger-de Groot at al. demonstrated that the invaginated ECs were located in the subendothelial region [40]. In addition, they found that a rat patent DA model exhibited an abundant subendothelial elastic lamina and failure of intimal formation [41]. Therefore, they suggest that some of the intimal-mesenchymal cells are also derived from the invaginated ECs [42]. In agreement with their idea, the present data suggest that EnMT-related signal pathways can be active in the DA endothelium and contribute to physiological intimal thickness of the DA.

MetaCore enrichment analysis also revealed that the retinol metabolism pathway worked in the DA ECs. The key genes lecithin∶retinol acyltransferase (Lrat), aldehyde dehydrogenase 1 family, member A1 (Aldh1a1), and Aldh1a7 are listed in the pathway. It is noteworthy that the endogenous retinol signaling may play a role in inducing and maintaining smooth muscle differentiation in the DA [43] and that maternal vitamin A accelerates vascular maturation of the DA in premature fetuses, at least in terms of vascular contraction [44], [45]. Furthermore, our previous study showed that maternally administrated vitamin A increased fibronectin production and intimal thickening in the rat DA [46]. Therefore, we may reconsider vitamin A as a candidate for treatment of patent DA, even though one study demonstrated that postnatal vitamin A therapy did not have a beneficial effect on ductal closure in premature infants [47]. Indeed, there are several studies demonstrating that vitamin A induces various embryonic developments via many different pathways such as Tgfb2, Cdh2, or Pitx2 [48]. Recently, Amengual at al. identified that Lrat is critical for cellular uptake of vitamin A from serum retinol-binding protein [49].

In conclusion, the present comprehensive transcription analysis identified the novel DA endothelium-dominant genes during a perinatal period that are highly related with biological processes involved in morphogenesis and development. Moreover, we found that regulation of epithelial-to-mesenchymal transition, cell adhesion, and retinol metabolism are the active pathways that form DA-specific endothelium. Newly identified DA endothelium-dominant genes may play an important role in DA-specific functional and morphologic characteristics.

## Supporting Information

Figure S1 **The representative figures of FACS gating strategy.** Cell debris and doublets were removed by light scattering; forward-scattered light (FSC) and side-scattered light (SSC). FSC and SSC are the parameter of cell-surface area/size and cell-internal complexity, respectively. A. The primary gating was done by removing the factors that affected FSC- and SSC- area. B. The secondary gating with FSC-height and width. C. The third gating with SSC-height and width. D. After those three gating steps by light scattering, dead cells were detected and removed by propidium iodide (PI) staining. E. Population of cells reacted with FITC-conjugated anti-CD31 antibody and APC-Cy7-conjugated anti-CD45 antibody. F. Population of cells reacted with fluorescence conjugated anti-control IgG antibodies to confirm nonspecific binding of antibodies.
(TIFF)

Figure S2 **Validation using quantitative RT-PCR.**
(TIFF)

Table S1 **The genes that have** ***p*** **<0.001 but range between 0.5< Fold change<2.0 in F.**
(DOCX)

Table S2 **The genes that have** ***p*** **<0.001 but range between 0.5< Fold change<2.0 in N.**
(DOCX)
