# Correction: Transcription Profiles of Endothelial Cells in the Rat Ductus Arteriosus during a Perinatal Period

**DOI:** 10.1371/annotation/ebd2da91-3f5a-4b50-8f16-9d7150795c69

**Published:** 2014-01-06

**Authors:** Norika Mengchia Liu, Tomohiro Yokota, Shun Maekawa, Ping Lü, Inbun Tei, Hideki Taniguchi, Utako Yokoyama, Takashi Kato, Susumu Minamisawa

The name of the fifth author is incorrect. The correct name is: Yun-Wen Zheng. The correct Citation is: Liu NM, Yokota T, Maekawa S, Lü P, Zheng Y-W, et al. (2013) Transcription Profiles of Endothelial Cells in the Rat Ductus Arteriosus during a Perinatal Period. PLoS ONE 8(9): e73685. doi:10.1371/journal.pone.0073685. The correct abbreviation of the fifth author's name in the Author Contributions Statement is: YWZ. 

