# Supplementary figures and images for: Transcription Profiles of Endothelial Cells in the Rat Ductus Arteriosus during a Perinatal Period
Source: PLoS One. 2013 Sep 27;8(9):e73685. doi: 10.1371/journal.pone.0073685 (PMC3785468; doi:10.1371/journal.pone.0073685)

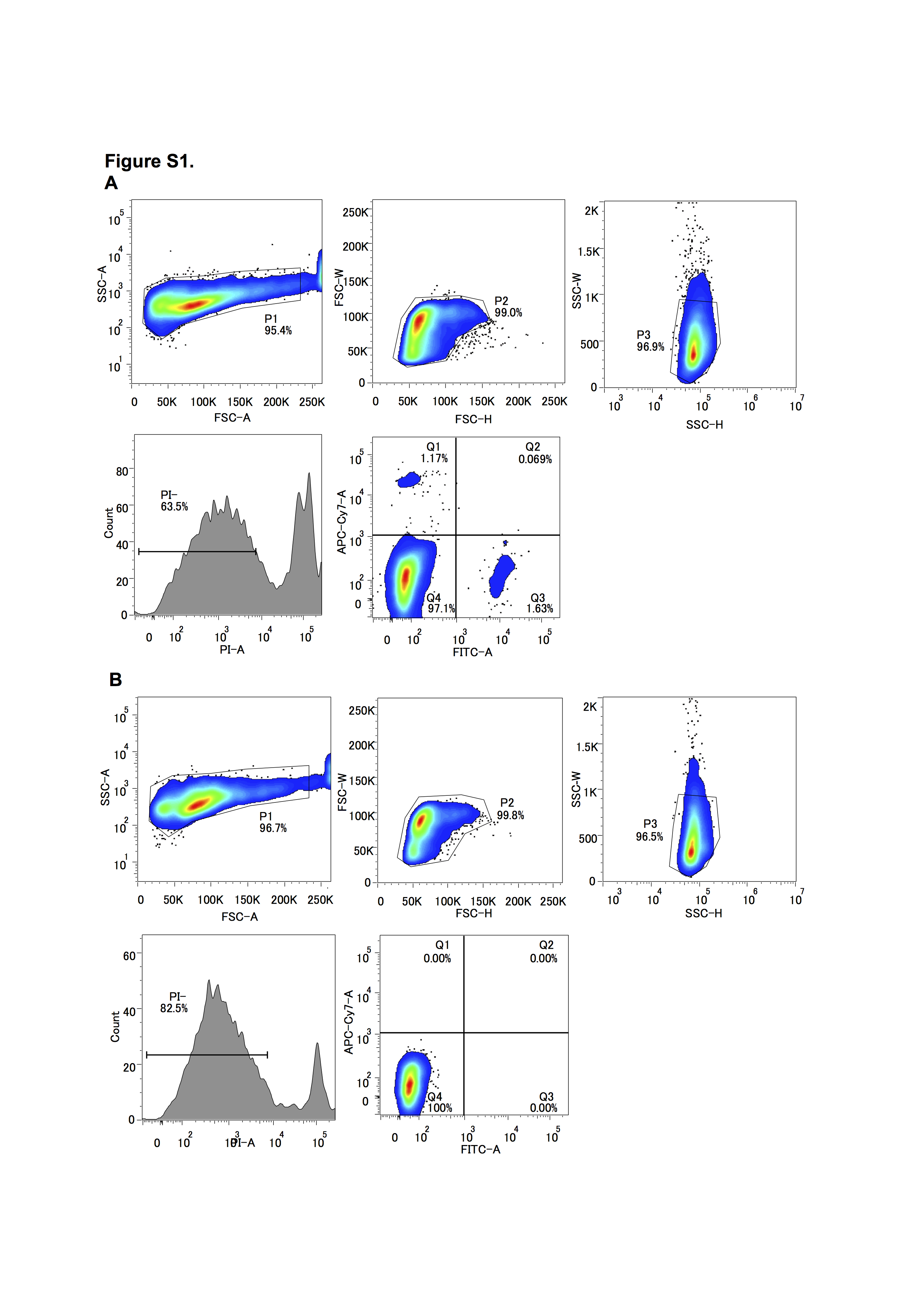

Supplement: Figure S1 — The representative figures of FACS gating strategy. Cell debris and doublets were removed by light scattering; forward-scattered light (FSC) and side-scattered light (SSC). FSC and SSC are the parameter of cell-surface area/size and cell-internal complexity, respectively. A. The primary gating was done by removing the factors that affected FSC- and SSC- area. B. The secondary gating with FSC-height and width. C. The third gating with SSC-height and width. D. After those three gating steps by light scattering, dead cells were detected and removed by propidium iodide (PI) staining. E. Population of cells reacted with FITC-conjugated anti-CD31 antibody and APC-Cy7-conjugated anti-CD45 antibody. F. Population of cells reacted with fluorescence conjugated anti-control IgG antibodies to confirm nonspecific binding of antibodies. (TIFF) [file pone.0073685.s001.tiff]

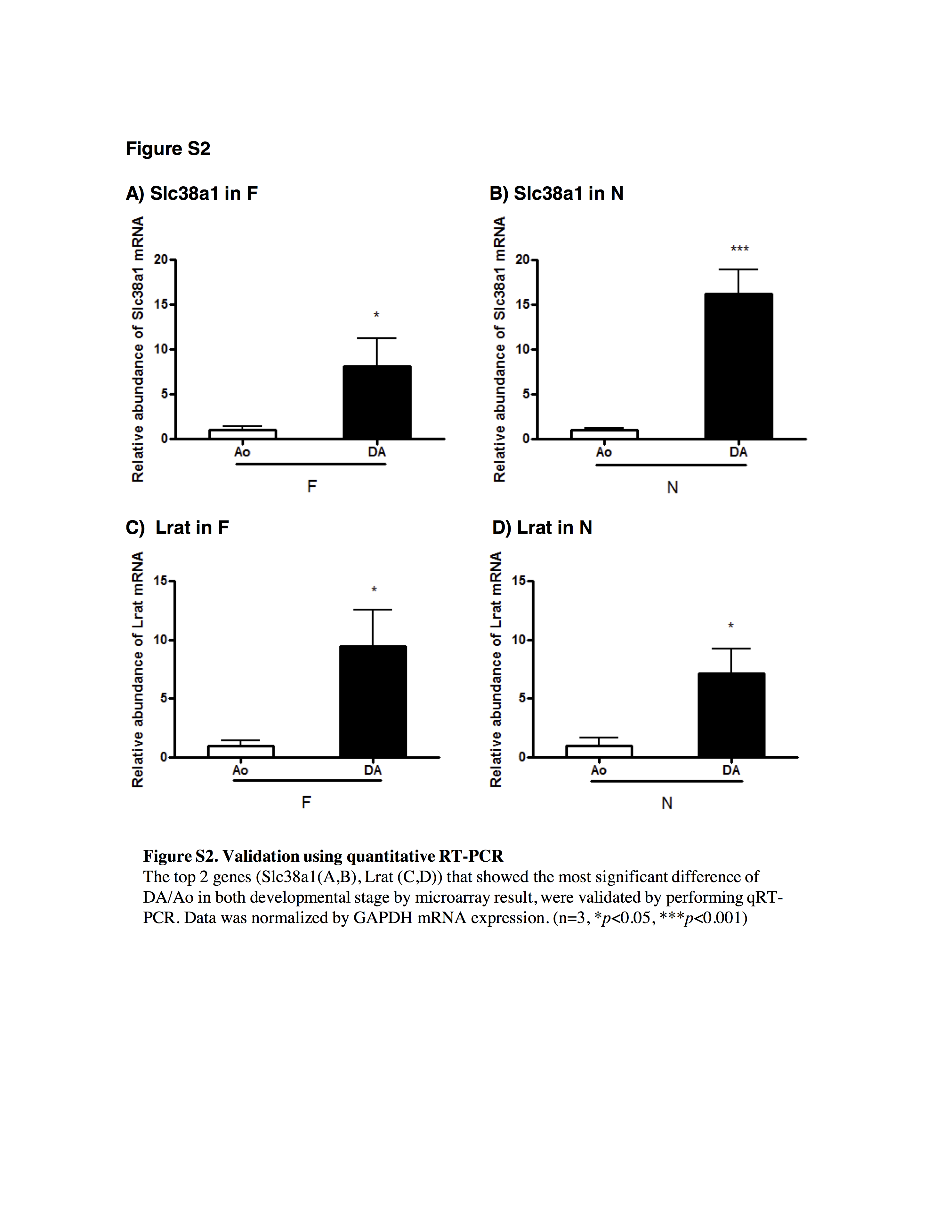

Supplement: Figure S2 — Validation using quantitative RT-PCR. (TIFF) [file pone.0073685.s002.tiff]
