# Supplementary material for: Transcription Profiles of Endothelial Cells in the Rat Ductus Arteriosus during a Perinatal Period
Source: PLoS One. 2013 Sep 27;8(9):e73685. doi: 10.1371/journal.pone.0073685 (PMC3785468; doi:10.1371/journal.pone.0073685)
Supplement: Table S1 — The genes that have p <0.001 but range between 0.5< Fold change<2.0 in F. (DOCX) [file pone.0073685.s003.docx]

**Table S1** **The genes that have *p*<0.001 but range between 0.5< Fold change<2.0 in F**

| Probe | mRNA | Gene | Fold change | *p* value |
| --- | --- | --- | --- | --- |
| set ID | Description | Symbol | DA/Ao | DA vs Ao |
| 10703144 | ribosomal protein S6 kinase polypeptide 2 | Rps6ka2 | 0.52 | 2.54E-06 |
| 10847260 | protein tyrosine phosphatase, receptor type, J | Ptprj | 0.52 | 9.76E-04 |
| 10735578 | ATPase, Ca++ transporting, ubiquitous | Atp2a3 | 0.54 | 2.38E-04 |
| 10899354 | activin A receptor type II-like 1 | Acvrl1 | 0.55 | 5.88E-04 |
| 10769349 | flavin containing monooxygenase 4 (Fmo4), transcript variant 2 | Fmo4 | 0.56 | 5.93E-04 |
| 10875282 | pleiomorphic adenoma gene 1 | Plag1 | 0.56 | 6.88E-04 |
| 10767565 | major facilitator superfamily domain containing 4 | Mfsd4 | 0.57 | 4.40E-04 |
| 10796564 | plexin domain containing 2 | Plxdc2 | 0.57 | 4.18E-04 |
| 10870713 | similar to Low-density lipoprotein receptor-related protein 8 precursor | Lrp8 | 0.59 | 6.54E-04 |
| 10713382 | phosphorylase, glycogen, muscle | Pygm | 0.60 | 3.10E-04 |
| 10717891 | ezrin | Ezr | 0.60 | 2.91E-04 |
| 10785590 | Kruppel-like factor 12 | Klf12 | 0.63 | 1.53E-04 |
| 10902446 | CCR4-NOT transcription complex, subunit 2 | Cnot2 | 0.63 | 4.02E-04 |
| 10819770 | EGF, latrophilin and seven transmembrane domain containing 1 | Eltd1 | 0.64 | 4.01E-04 |
| 10849707 | mal, T-cell differentiation protein-like | Mall | 0.65 | 9.22E-05 |
| 10743914 | similar to chromodomain helicase DNA binding protein 3 isoform 3 | Chd3 | 0.66 | 5.85E-05 |
| 10866988 | similar to ARG99 homolog | Tmtc1 | 0.66 | 4.77E-04 |
| 10807272 | hydroxysteroid 11-beta dehydrogenase 2 | Hsd11b2 | 0.68 | 7.53E-04 |
| 10777350 | cytokine like 1 | Cytl1 | 0.71 | 6.74E-04 |
| 10768834 | xenotropic and polytropic retrovirus receptor 1 | Xpr1 | 0.71 | 4.54E-04 |
| 10782609 | protein tyrosine phosphatase, receptor type, G | Ptprg | 0.71 | 1.02E-04 |
| 10886252 | thyroid stimulating hormone receptor | Tshr | 0.72 | 9.10E-04 |
| 10940622 | potassium channel tetramerization domain containing 10 | --- | 0.77 | 7.17E-04 |
| 10799942 | similar to Rho GTPase activating protein 21 | Arhgap21 | 0.77 | 6.10E-04 |
| 10748314 | similar to inositol-requiring 1 alpha | Ern1 | 0.78 | 9.24E-04 |
| 10710695 | trinucleotide repeat containing 6a | Tnrc6a | 0.85 | 2.36E-04 |
| 10738591 | frizzled homolog 2 (Drosophila) | Fzd2 | 1.29 | 7.35E-04 |
| 10940688 | fibroblast growth factor 18 | --- | 1.46 | 6.39E-04 |
| 10765285 | similar to G protein-coupled receptor 161 isoform 2 | Gpr161 | 1.46 | 3.37E-04 |
| 10927692 | four and a half LIM domains 2 | Fhl2 | 1.68 | 7.76E-04 |
| 10774015 | AE binding protein 1 | Aebp1 | 1.74 | 5.77E-04 |
| 10919103 | malic enzyme 1, NADP(+)-dependent, cytosolic | Me1 | 1.76 | 4.65E-04 |
| 10835292 | frequenin homolog (Drosophila) | Freq | 1.84 | 8.26E-05 |
| 10832782 | transmembrane protein 26 | Tmem26 | 1.90 | 1.41E-05 |

Thirty four genes shows a significant difference by *p* values (*p*<0.001) between DA and Ao though their fold change were neither more than 2 or less than 0.5 in F group. F: fetuses before breathing.
