# Supplementary material for: Transcription Profiles of Endothelial Cells in the Rat Ductus Arteriosus during a Perinatal Period
Source: PLoS One. 2013 Sep 27;8(9):e73685. doi: 10.1371/journal.pone.0073685 (PMC3785468; doi:10.1371/journal.pone.0073685)
Supplement: Table S2 — The genes that have p <0.001 but range between 0.5< Fold change<2.0 in N. (DOCX) [file pone.0073685.s004.docx]

**Table S2 The genes that have *p*<0.001 but range between 0.5< Fold change<2.0 in N**

| Probe | mRNA | Gene | Fold change | *p* value |
| --- | --- | --- | --- | --- |
| set ID | Description | Symbol | DA/Ao | DA vs Ao |
| 10745637 | matrix metallopeptidase 28 | Mmp28 | 0.53 | 7.63E-04 |
| 10703144 | ribosomal protein S6 kinase polypeptide 2 | Rps6ka2 | 0.55 | 9.72E-04 |
| 10847260 | protein tyrosine phosphatase, receptor type, J | Ptprj | 0.57 | 4.22E-04 |
| 10769621 | uridine-cytidine kinase 2 | Uck2 | 0.57 | 8.63E-04 |
| 10735578 | ATPase, Ca++ transporting, ubiquitous | Atp2a3 | 0.59 | 1.16E-04 |
| 10810570 | exocyst complex component 3-like | Exoc3l | 0.62 | 6.86E-04 |
| 10731025 | similar to Protein KIAA1914 | Afap1l2 | 0.63 | 6.10E-04 |
| 10889388 | ring finger protein 144A | Rnf144a | 0.64 | 5.93E-04 |
| 10829993 | platelet receptor Gi24 | MGC112715 | 0.65 | 7.63E-05 |
| 10716803 | utrophin | Utrn | 0.65 | 3.41E-04 |
| 10903459 | Kruppel-like factor 10 | Klf10 | 0.66 | 2.78E-04 |
| 10918092 | protein inhibitor of activated STAT, 1 | Pias1 | 0.71 | 8.95E-04 |
| 10815636 | similar to muscleblind-like 1 | Mbnl1 | 0.75 | 5.52E-04 |
| 10897096 | gasdermin D | Gsdmd | 0.77 | 5.34E-04 |
| 10785964 | dedicator of cytokinesis 9 | Dock9 | 0.77 | 5.62E-04 |
| 10777928 | LIM domain kinase 2 | Limk2 | 0.78 | 6.38E-04 |
| 10906882 | adenylate cyclase 6 | Adcy6 | 0.84 | 5.13E-04 |
| 10938446 | melanoma antigen, family D, 1 | Maged1 | 1.12 | 8.38E-04 |
| 10935928 | ribosomal protein L28 | Rpl28 | 1.19 | 8.88E-04 |
| 10724967 | dickkopf homolog 3 (Xenopus laevis) | Dkk3 | 1.22 | 3.16E-04 |
| 10820282 | versican (Vcan), transcript variant 1 | Vcan | 1.40 | 3.52E-05 |
| 10871540 | leucine proline-enriched proteoglycan (leprecan) 1 | Lepre1 | 1.42 | 8.75E-04 |
| 10728164 | protein phosphatase 2, regulatory subunit B', beta isoform | Ppp2r5b | 1.54 | 7.22E-04 |
| 10908751 | opioid binding protein/cell adhesion molecule-like (Opcml) | Opcml | 1.66 | 8.75E-04 |
| 10905988 | similar to Cadherin EGF LAG seven-pass G-type receptor 1 precursor | Celsr1 | 1.87 | 6.13E-04 |
| 10880163 | similar to CG9047-PA, isoform A | Nkain1 | 1.89 | 9.17E-05 |
| 10912174 | ADAM metallopeptidase with thrombospondin type 1 motif, 7 | Adamts7 | 1.94 | 6.91E-04 |

Twenty seven genes shows a significant difference by *p* values (*p*<0.001) between DA and Ao though their fold change were neither more than 2 or less than 0.5 in N group. N: neonates obtained 30 minutes after breathing.
